# Congenital syphilis unusually presenting with prematurity-related severe neonatal morbidities including meconium obstruction: A case report and review of the literature

**DOI:** 10.1097/MD.0000000000022321

**Published:** 2020-10-02

**Authors:** Hyun-Seung Lee, Jong In Lee, Jihyun Jeon

**Affiliations:** a Department of Pediatrics; b Pediatric surgery, CHA Gangnam Medical Center, CHA University School of Medicine, Seoul, Korea.

**Keywords:** congenital syphilis, intestinal obstruction, meconium, newborn, prematurity

## Abstract

**Rationale:**

Congenital syphilis (CS) can manifest as a variety of clinical presentations according to the severity in symptomatic infants during neonatal period. Preterm neonates with CS may have more clinical evidences of infection and be more severely affected by CS compared with term ones. With increasing survival of markedly premature infants for recent decades, CS may be a challenging problem in those with severe manifestations associated with combined pathophysiologies of CS and prematurity.

**Patient concerns:**

A very low birth weight infant at 32 weeks gestation presented with an unusual CS presentation consisting of prematurity-associated severe neonatal morbidities including meconium obstruction, prolonged cholestatic jaundice with elevated liver enzymes, and disseminated intravascular coagulation with a bleeding diathesis, in addition to common or typical manifestations of CS.

**Diagnoses:**

Congenital syphilis.

**Interventions:**

Therapy consisting of a complete course of parenteral penicillin, blood component therapy, proximal ileotomy with inspissated meconium evacuation and distal loop ileostomy, and other conservative treatments.

**Outcomes:**

Resolution with normal gastrointestinal function and improved liver function was observed.

**Lessons:**

This case suggests that in premature infants CS may manifest as unusual severe neonatal morbidities that may result from combination of syphilitic pathologies and contributors or conditions associated with prematurity including multisystem immaturity.

## 1 Introduction

Congenital syphilis (CS) is a fetal or child infection with *Treponema pallidum* from maternal syphilis via transplacental hematogenous transmission at any time in gestation or intrapartum direct inoculation by contacting with primary lesions.^[1]^ While the majority of CS cases are clinically silent at birth, symptomatic newborn cases may present with a wide range of clinical features depending on the severity due to treponemal multisystem involvement.[^1^,^2^] Preterm neonates may be more severely affected by CS than term ones.^[3]^ In the literature, severe neonatal morbidities such as meconium obstruction and disseminated intravascular coagulation (DIC) as CS manifestations have rarely been found, and largely in prematurely born infants with CS.[^4^,^5^] Herein, we report a very low birth weight premature infant with an unusual CS presentation consisting of prematurity-associated severe neonatal morbidities including meconium obstruction, persistent cholestatic jaundice, and DIC.

## 2 Case report

A Korean female infant with a weight of 1460 g (27th percentile) was born to a 40-year-old primigravida primipara woman at 32 weeks’ gestation by an emergency Cesarean section for fetal distress. The mother had a history of a negative screening test 6 months before delivery and no treatment for syphilis. Apgar scores were 5 and 7 at 1 and 5 minutes, respectively. Physical examination revealed a desquamative rash on the hands and feet, distended abdomen, palpable hepatosplenomegaly, and jaundice. Laboratory data demonstrated hemolytic anemia (hemoglobin 10.2 g/dl, hematocrit 32.7%, and reticulocyte 7.86%), leukocytosis (white blood cells [WBC] 17,630/μl), elevated C-reactive protein (9.39 mg/dl), and hypoglycemia (30 mg/dl). Rapid plasma reagin and fluorescent treponemal antibody absorption tests on the neonate and mother were all reactive. Cerebrospinal fluid analysis disclosed a Venereal Disease Research Laboratory (VDRL) titer at 1:32, a WBC of 7/μl, and a protein level of 230 mg/dl. Diffuse osteochondritis was identified on long-bone radiographs. A course of intravenous aqueous penicillin G (50,000 units/kg/dose, every 12 hours for 1 week and thereafter every 8 hours for a total of 3 weeks) was established. Because of poor respiratory effort with undersized lungs at birth, the infant was mechanically ventilated for 17 days, with transient episodes of respiratory distress syndrome and pneumonitis.

Persistent bleeding from venipuncture sites and hemorrhagic endotracheal aspirates were noted on postnatal day 1. DIC was identified on the hematological profile (platelet count, 20,000/μl; prothrombin time, 16.5 vs control, 10.1 to 12.6 seconds with the international normalized ratio of 1.46 vs 0.93–1.13; partial thromboplastin time, 69.3 vs 23.6 to 31.1 seconds; antithrombin III, 14%; fibrinogen, 136 mg/dl; and D-dimer, 16511.3 ng/ml DDU). Serial cranial ultrasound revealed both intraventricular hemorrhage, up to grade 2. Blood component therapy comprising fresh frozen plasma, antithrombin III, and platelet concentrates corrected the hematological indices of coagulopathy and the bleeding diathesis.

Initial liver function tests revealed an elevated total bilirubin (6.3 mg/dl) with the direct fraction of 73% (4.6 mg/dl) and raised serum aspartate aminotransferase (AST) and alanine aminotransferase (ALT) (355 and 97 IU/L, respectively) (Table 1). After the initiation of penicillin therapy, the direct hyperbilirubinemia was progressive until postnatal day 4, reflecting the hemolysis at birth along with initial high AST levels (Table 1). Serum ALT and AST levels were elevated in response to daily administered penicillin dosage (Table 1). After a complete course of parenteral penicillin, serum bilirubin, ALT and AST reduced to the initial high levels (in AST, the level on day 3), which gradually improved thereafter but direct hyperbilirubinemia persisted (Table 1). Cholescintigraphy (DISIDA scan) on day 67 depicted biliary-to-bowel transit on delayed 6-hour images.

**Table 1:**
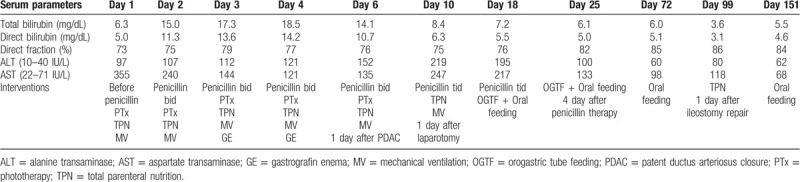
Laboratory data of liver function tests in the present case.

**Figure 1:**
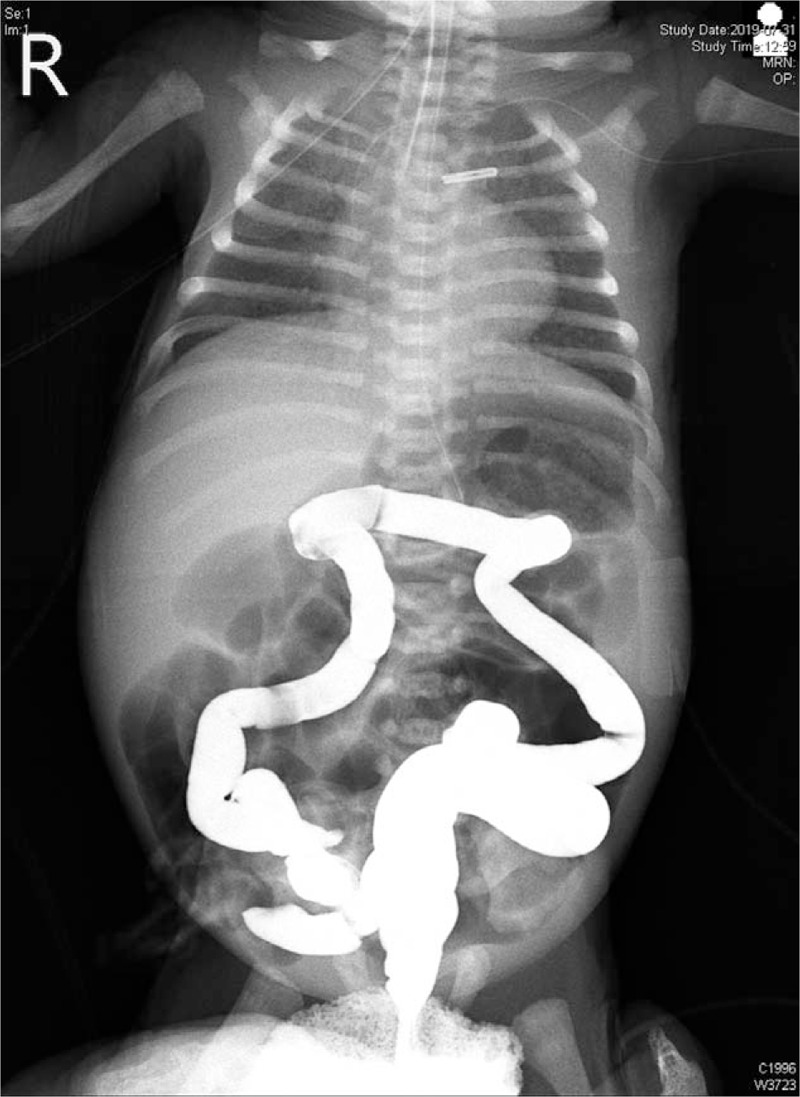
Water-soluble contrast enema on postnatal day 9 demonstrates a typical microcolon and no reflux into the distal ileum. Multiple, diffusely and uniformly dilated loops of small bowel is also noted with massive abdominal distension.

On postnatal day 3, small bowel obstruction was suspected because serial abdominal radiographs showed progressive gastric dilatation with a centrally located small bowel gas and no colon gas. The infant spontaneously passed an average amount of meconium defecating ten times for the first 2 days. Total parenteral nutrition was instituted. Gastrografin enemas were performed every 24 hours for 5 days (on days 3–9 except days 5 and 6 of patent ductus arteriosus ligation and the first postoperative day) and produced small amounts of stool. Serial contrast enemas consistently demonstrated a microcolon with multiple intraluminal filling defects (meconium plugs) and no possible reflux into the distal ileum, with progressively worsening gastric and proximal bowel loops dilation (Fig. 1). On day 9, at laparotomy two-thirds of the small bowel was plugged by inspissated meconium, which was not milked distally and located in the bowel segment 80 cm long (from the jejunum 10 cm from the ligament of Treitz to the ileum 50 cm from the ileocecal valve). After proximal ileotomy at 70 cm distance from the ileocecal valve and warm saline irrigation, most of the dark green and jelly-like sticky meconium was evacuated and distal loop ileostomy was performed.

Serological assays for toxoplasmosis, rubella, cytomegalovirus, herpes, and human immunodeficiency virus were negative. Testing for thyroid functions and alpha-1 antitrypsin levels, genetic analysis for cystic fibrosis, and submucosal rectal biopsies were all within normal range.

Postoperative follow-up was uneventful. Enteral feeding was initiated on day 14 and full oral feeding was reached on day 35. The repeated cerebrospinal fluid VDRL test on day 87 was nonreactive. The ileostomy was repaired on day 98.

This study was approved by the Institutional Review Board (IRB) of CHA Gangnam Medical Center (IRB No. GCI-20-06). The patients legal guardian provided a written informed consent for publication of this case report and accompanying images.

## 3 Discussion

Our infant with CS and prematurity with a very low birth weight presented at birth with well-recognized typical clinical, laboratory, and radiographic features of CS as listed in Table 2. Moreover, the infant showed CS manifestations rarely found in the literature, including meconium obstruction, prolonged cholestatic jaundice with elevated liver enzymes, and DIC with intrapulmonary and intraventricular hemorrhages. However, these unusual CS presentations can also be found in critically ill preterm infants, particularly those born very early and necessitating neonatal intensive care.

**Table 2:**
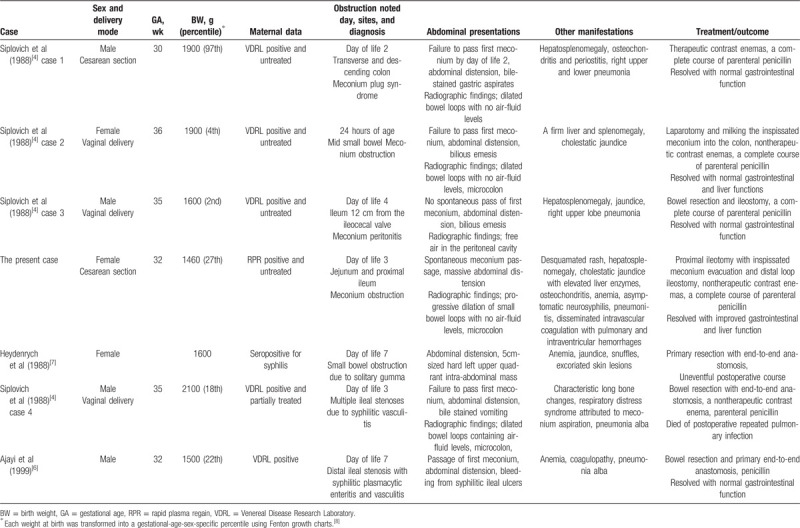
Overview of the published congenital syphilis infants and our infant with meconium obstruction (the first 4 cases) and other intestinal obstructions.

Table 2 depicts the current case and the published cases of neonatal CS presenting with meconium obstruction of various severities (the first 4 cases)^[4]^ and other intestinal obstructions caused by ileal stenosis due to syphilitic enteritis and vasculitis[^4^,^6^] and an intraabdominal gumma penetrating small bowel loops.^[7]^ All cases were premature infants with low birth weight (<2500 g) and other various CS signs. All had relatively low age-sex-specific birthweight percentiles^[8]^ and small bowel obstruction, except 1 with benign meconium plugs in the large bowel and the 97th birthweight percentile despite the earliest gestational age, treated with contrast enemas. Two cases including our infant (with 4th and 27th birthweight percentiles, respectively) had mid small bowel meconium obstruction, which is a higher level than the most frequent obstruction site distal ileum, and required surgical intervention with nontherapeutic contrast enemas. One with meconium peritonitis showed the lowest birthweight percentile. These suggest that the severity of syphilitic meconium obstruction might be associated with birthweight percentile.

Meconium obstruction is neonatal intestinal obstruction by inspissated meconium encompassing a variety of clinical syndromes.[^9^,^10^] Two entities of meconium obstruction have largely been reported:

1. meconium ileus associated with cystic fibrosis (defined as terminal ileal obstruction with failure to pass meconium within 48 hours of birth and usually require surgical care);[^10^,^11^] and
2. meconium disease related to prematurity and low birth weight without cystic fibrosis and Hirschsprungs disease[^9^,^12^] (characterized by low grade obstruction following spontaneous first meconium passage mostly responding to conservative approach or water-soluble contrast enemas,^[9]^ despite debatable reported cases such as cases in mature infants and meconium ileus-like cases).[^10^,^13^]

Basically, various meconium obstruction syndromes have common underlying mechanisms related to intestinal hypomotility and/or abnormal highly viscid and adherent meconium production during pregnancy.[^11^,^12^,^13^] In our case, the combined following mechanisms of CS and prematurity may be involved in the development of meconium obstruction. Chronic inflammation of CS may contribute to meconium obstruction via cooperation of intestinal motility disturbance due to syphilitic enterocolitis and inspissated meconium formation secondary to exocrine pancreatic insufficiency due to syphilitic pancreatitis.^[4]^ Mechanisms associated with prematurity may include immaturity of the intestinal nervous system, intestinal hypoperfusion, and abnormal tenacious mucous production by intestinal goblet cells.[^10^,^12^,^13^]

The persistent neonatal cholestasis in our case may be a result of the common intersection of multiple factors including syphilitic hepatitis, nonsyndromatic paucity of intrahepatic bile ducts,^[14]^ immaturity of the newborn liver, intestinal failure because of meconium obstruction, and total parenteral nutrition.^[15]^ The penicillin therapy aggravated the liver dysfunction from syphilitic hepatitis, as shown by serum ALT levels specifically responding to penicillin dose changes (Table 1). The DIC may result from syphilitic vasculitis and syphilitic involvement of liver (hepatitis) and bone marrow (thrombocytopenia)^[16]^ along with the immature coagulation and fibrinolytic systems with little reserve capacity in the preterm newborn state to be susceptible to this systemic thrombohemorrhagic disorder.^[17]^

Consequently our case suggests that in premature infants CS may manifest as unusual severe neonatal morbidities that may result from combination of syphilitic pathologies and contributors or conditions associated with prematurity including multisystem immaturity. Hence, clinicians should consider this peculiar clinical aspect of neonatal CS that is not shown in sexually transmitted syphilis, particularly in premature infants affected with CS.

## Author contributions

**Conceptualization:** Hyun-Seung Lee, Jihyun Jeon.

**Data curation:** Hyun-Seung Lee, Jong In Lee.

**Formal analysis:** Hyun-Seung Lee.

**Investigation:** Hyun-Seung Lee.

**Project administration:** Hyun-Seung Lee.

**Supervision:** Jong In Lee, Jihyun Jeon.

**Validation:** Jihyun Jeon.

**Visualization:** Jong In Lee.

**Writing – original draft:** Hyun-Seung Lee.

**Writing – review & editing:** Jong In Lee, Jihyun Jeon.
